# The Preparation and Properties of Composite Hydrogels Based on Gelatin and (3-Aminopropyl) Trimethoxysilane Grafted Cellulose Nanocrystals Covalently Linked with Microbial Transglutaminase

**DOI:** 10.3390/gels8030146

**Published:** 2022-02-26

**Authors:** Shouwei Zhao, Zhiwei Chen, Yaqi Dong, Wenhui Lu, Deyi Zhu

**Affiliations:** Faculty of Light Industry, Qilu University of Technology (Shandong Academy of Sciences), Jinan 250353, China; zsw18834407603@163.com (S.Z.); w36000@163.com (Z.C.); dyq15628875660@163.com (Y.D.); whlu@qlu.edu.cn (W.L.)

**Keywords:** gelatin-based composite hydrogels, cellulose nanocrystals, silanization, mechanical property, microbial transglutaminase

## Abstract

Mechanically enhanced gelatin-based composite hydrogels were developed in the presence of functionalized cellulose nanocrystals (CNCs) employing microbial transglutaminase (mTG) as a binding agent. In this work, the surfaces of CNCs were grafted with (3-Aminopropyl) trimethoxysilane with a NH_2_ functional group, and the success of CNCs’ modification was verified by FTIR spectroscopy and XPS. The higher degree of modification in CNCs resulted in more covalent cross-linking and dispersibility within the gelatin matrix; thus, the as-prepared hydrogels showed significantly improved mechanical properties and thermo-stability, as revealed by dynamic rheological analysis, uniaxial compression tests and SEM. The biocompatibility of the obtained hydrogels was evaluated by the MTT method, and it was found that the grafted CNCs had no obvious inhibitory effect on cell proliferation. Hence, the mechanically enhanced gelatin-based hydrogels might have great potential in biomedical applications.

## 1. Introduction

Hydrogels are a class of 3D network materials that can exhibit the ability to swell and retain a large amount of water within their networks through surface tension and capillary forces, but they will not dissolve in water. Thanks to their high water content, they also possess flexibility similar to natural tissues [1]. These characteristics enable hydrogels to be widely used in the field of biomedicine, including tissue-engineering scaffolds [2], drug-delivery systems [3], tissue adhesive [4], and controlled release [5,6]. Hydrogels can be derived from natural polymers and synthetic polymers. Although synthetic polymer-based hydrogels provide high mechanical properties and other interesting properties, natural polymers (chitosan, alginates, hyaluronic acid, cellulose, gellan gum, dextran, gelatin, etc.) derived hydrogels are more competitive due to their outstanding biocompatibility and biodegradability [7]. Amongst these natural polymers, gelatin is considered to be the most appropriate one. Gelatin is produced by partial hydrolysis of collagen, which is a fibrous protein forming a majority part of the extracellular matrix. Due to its low immunity, biocompatibility, biodegradability and multiple active sites, gelatin can be flexibly modified and has been widely applied in pharmaceutical and medical applications [8,9,10,11]. However, the weak mechanical properties of gelatin-based hydrogel greatly limit their potential for biomedical fields. These disadvantages can be reduced by cross-linking the materials [8].

To stabilize the gelatin structure by cross-linking, several methods have been proposed, including physical, chemical and enzymatic cross-linking. Physical cross-linking gelatin hydrogels were prepared by hydrophobic interaction [12], irradiation [13] and dehydrothermal treatment [6], which showed low toxicity and have been diversely applied in the biomedical field. Nevertheless, it is hardly to control the cross-linking density of the physical hydrogel, which greatly limits further improvement of mechanical properties [14]. Chemical cross-linking gelatin hydrogels were formed by covalent bonds with irreversible cross-linking, showing relatively high mechanical properties [11]. However, unreacted cross-linkers can cause cytotoxicity and induce inflammation [15]. Recently, enzymatic cross-linking has attracted considerable research interest because of its high specificity, mild reaction conditions and good cytocompatibility. For enzymatic cross-linking gelatin hydrogels, microbial transglutaminase (mTG) was commonly used to improve the biocompatibility and decrease cytotoxicity [10,16], which can catalyze the covalent cross-linking reaction between the γ-carboxamide group of glutamine residues and primary amines (most often the ε-amino group of lysine) by forming isopeptide bonds [17]. For instance, gelatin-laminin hydrogels have been prepared by enzymatical cross-linking a laminin layer on the top of gelatin hydrogels using mTG, aiming to establish an ideal research platform of multi-cellular tissues [18]. Yu and coworkers have found that cross-linking gelation by mTG can obviously enhance the thrombin hemostatic [19]. Broderick et al. have used mTG to prepare gelatin-based scaffolds, which showed excellent mechanical properties and thermal stability [20].

Moreover, introducing nanomaterials with good mechanical properties was another efficient method for enhancing the mechanical properties of gelatin-based hydrogels [21]. Based on that, nanocellulose with excellent mechanical strength, biocompatibility, biodegradability and environmentally friendliness has become one of the research hotspots for the preparation of gelatin hydrogels, such as oxidized nanowhiskers [22] and cellulose nanocrystals (CNCs) [23,24]. In our previous study, CNCs were used as a nanofiller to prepare gelatin/CNCs composite hydrogels (Gel-TG-CNCs) by the enzymatic cross-linking method. The as-prepared Gel-TG-CNCs proved to have excellent biocompatibility and improved mechanical properties [25]. However, only physical interaction between gelatin chain and CNCs was formed in Gel-TG-CNCs. We hypothesized that covalently cross-linking CNCs into the gelatin network will further improve the mechanical stability of the gelatin-based hydrogels. In the present work, CNCs were firstly modified by (3-aminopropyl) trimethoxysilane (APTMS), and amino groups were grafted on the surface of CNCs. In this way, CNCs can be connected to gelatin in the form of a covalent bond due to the catalyzation of mTG when the novel gelatin/cellulose nanocrystal composite hydrogel (Gel-TG-Si-CNCs) was formed. The mechanical properties of the obtained hydrogels were well characterized by rheological and compressive tests. The biocompatibility of the hydrogels was also evaluated in vitro.

## 2. Results and Discussion

### 2.1. FTIR Spectroscopy

**Figure 1:**
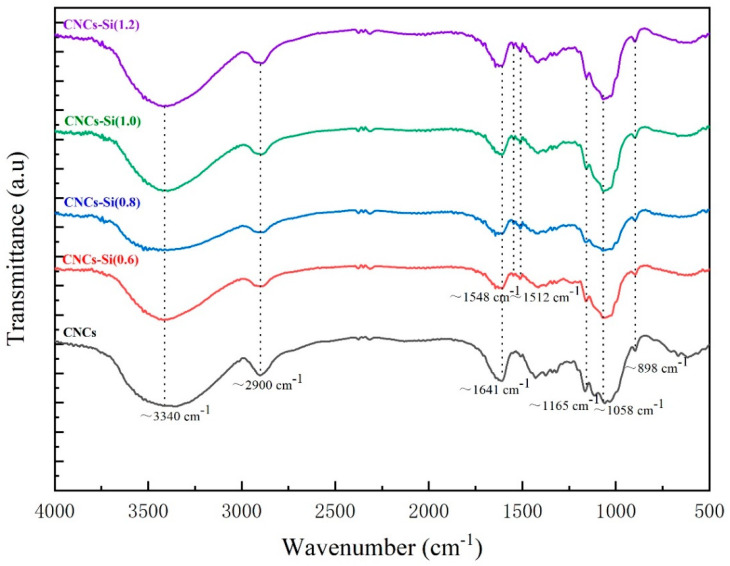
FTIR spectra of CNCs and CNCs-Si.

The modification of CNCs by APTMS is believed to be as follows. Because of the abundant free hydroxyl groups on the surface of CNCs, a hydrogen bond can be stably formed between silanols with CNCs. For the preparation of CNC–Si, a high temperature (110 °C) can further promote the condensation and formation of a polysiloxane layer onto CNCs. Figure 1 depicts the FTIR spectra of CNCs before and after modification with APTMS. The FTIR spectra of unmodified CNCs showed distinguish bands of 3340, 2900 and 1641 cm^−1^, which, respectively, attributed to O–H stretching vibrations, C–H stretching vibrations of alkane and asymmetric of CH_2_ stretching, and O–H deformation [26]. The C–C stretching at 1158 cm^−1^ and C–O stretching at 1058 cm^−1^ indicate the amorphous structure [27]. For the four kinds of CNCs-Si, the primary amine N–H stretch signal at 3500–3300 cm^−1^ was overlapped by O–H stretching vibrations. However, two peaks at 1548 and 1512 cm^−1^ (corresponding to –NH_2_ bending vibrations) clearly indicated the presence of primary amine groups on CNCs–Si. For CNCs, there was only one peak at 1641 cm^−1^, which came from the stretching vibration of C=C bonding [28]. The characteristic absorption peaks of Si–O–C and Si–O–Si formatted by condensation of the hydroxyl groups were difficult to be observed, attributed to the strong C–O–C vibration bands at 1058 cm^−1^ [29].

### 2.2. XPS

**Figure 2:**
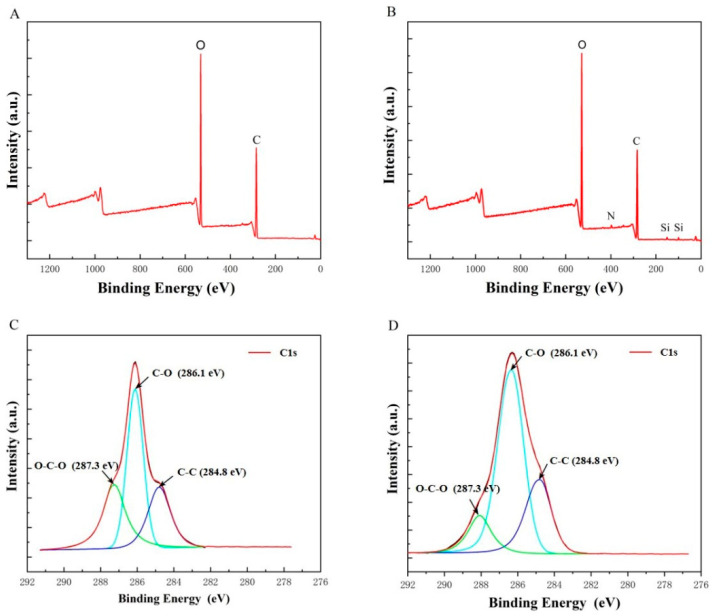
Survey XPS spectra of CNCs (**A**) and CNCs-Si (**B**); high-resolution XPS spectra of C_1s_ of CNCs (**C**) and CNCs-Si (**D**).

The XPS spectra of CNCs and CNCs-Si were shown in Figure 2, and the relative atomic concentration and oxygen/carbon ratios were tabulated in Table 1. For CNCs-Si, an N1s and two Si (Si_2s_, Si_2p_) peaks were detected, which were not observed for non-modified CNCs, indicating the possible presence of the bonding of silane onto CNCs. These changes are also identified in Table 1, in which the atomic concentration of N and Si of modified CNCs is 1.42% and 1.54%, respectively. In addition, the surface O/C ratio of CNCs and CNCs-Si were 0.66 and 0.64, respectively. According to the report by Lu et al. [29], this decrease in the O/C ratio during APTMS grafting was ascribed to the carbon-richer (propyl moiety) of APTMS. More convincing evidence for successful APTMS modification can be found from the high-resolution XPS spectra of the C_1s_ peak (Figure 2C, D). The deconvolution of the peak associated with carbon atoms in the XPS spectra of CNCs showed three types of carbon bonds: C–C (284.8 eV), C–O (286.1 eV) and O–C–O (287.3 eV) [23,30]. In Figure 2D, the intensity of the C–C peak increased with surface silanization, and the intensity of the C–C peak was much higher than that of the O–C–O peak, which was in contrast with results in Figure 2C. This change was mainly due to the presence of the propyl groups in APTMS. The XPS results corroborated the FTIR analyses further supported the idea that CNCs modification was successful.

### 2.3. Rheological Behavior

**Figure 3:**
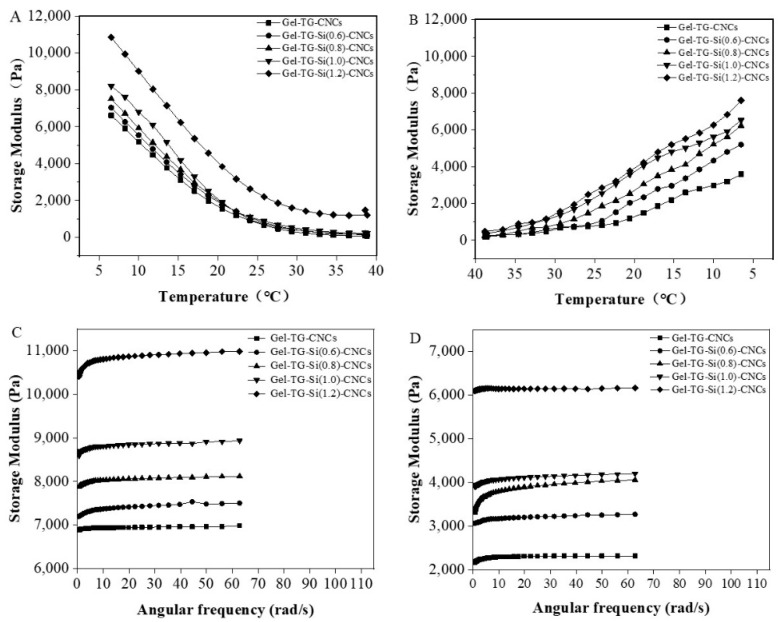
The storage modulus of the gelatin/CNCs hydrogels tested under temperature sweep (**A**): 5–40 °C and (**B**): 40–5 °C and tested under frequency sweep at 5 °C (**C**) and 15 °C (**D**).

The material stiffness was regularly evaluated from the storage modulus (G′), which reflected hydrogels elasticity or strength and a higher G′ value indicated higher strength. To evaluate the hydrogel mechanical stability, we performed variable temperature rheological analysis of the gelatin/silane-modified CNCs hydrogels in the following experiment. Figure 3A,B clearly showed the G′ values of the Gel-TG-Si-CNCs and Gel-TG-CNCs hydrogels in the temperature range of 5–40 °C and 40–5 °C, respectively. The hydrogels exhibited thermo-reversible behavior and a general trend was that G′ declined with the increase in temperature. In the presence of mTG, chemical-physical gels were formed, where the physical networks (self-assembly triple helix, hydrogen bonding and hydrophobic linkages) have cooperated with covalent cross-linking [25,31] to maintain the stability of the hydrogel. With the increasing temperature, the collagen triple helix unfolding was activated and other physical cross-linking interactions became progressively weaker. It resulted in the decreasing of the storage modulus. As expected, Gel-TG-Si-CNCs samples showed enhanced storage modulus. In particular, at lower temperatures (5 °C), G′ of Gel-TG-Si (1.2)-CNCs reached about 11,000 Pa, which was 1.6 times higher than that of Gel-TG-CNCs. Additionally, at higher temperatures (25–40 °C), the enhancement was more pronounced. This can be ascribed to the presence of more covalent cross-linking within gelatin chains and modified CNCs, which enhanced the thermo-stability of the gel. Moreover, silylated CNCs had increased dispersibility in gelation solution in comparison with the original one [32], which tended to produce stronger reinforcing effects on hydrogels with improved storage modulus.

Further frequency sweep tests were carried out in the range of 0.0628–62.8 rad/s at 0.5% strain. The G′ values of the hydrogel samples at 5 °C and 15 °C are presented separately in Figure 3C,D. Herein, G′ was a measure of the energy stored in the material and recovered from it per cycle during the shear process. For all samples, G′ increased with increasing angular frequency and exhibited a plateau over the higher frequency range, which indicated high structural stability [33]. The gelatin/CNCs-Si cross-linking effect was shown by the significant increase in G′ found for Gel-TG-Si-CNCs compared with Gel-TG-CNCs. In the case of Gel-TG-Si (1.2)-CNCs, the storage modulus was about three times higher than that of Gel-TG-CNCs at 15 °C. Overall, within the investigated scope the higher degree of modification of CNCs led to a significantly higher mechanical stability for the gelatin-based hydrogels. The results of rheological behavior confirmed our hypothesis proposed above that covalently cross-linking CNCs into the gelatin network will further improve the mechanical stability of the gelatin-based hydrogels.

### 2.4. Gel Strength

**Figure 4:**
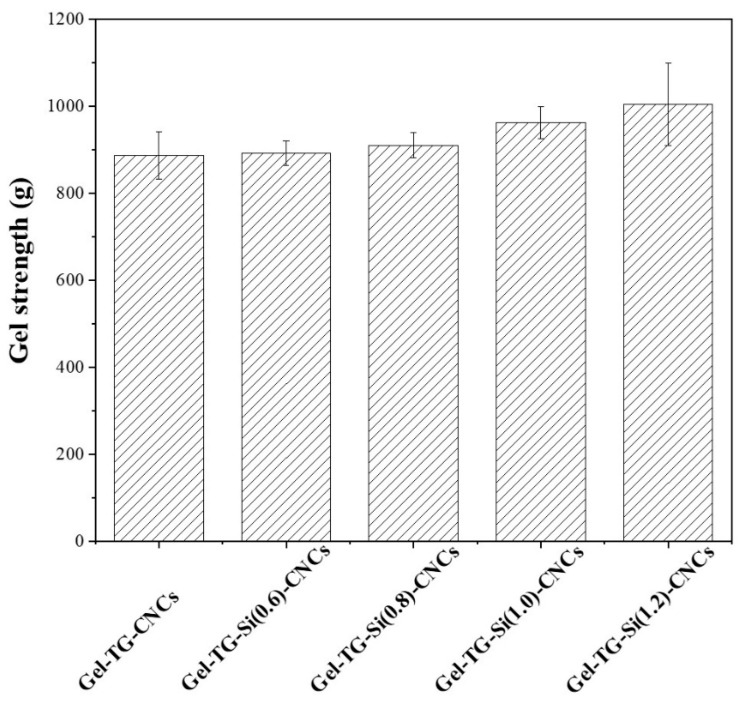
The gel strength of gelatin/CNCs hydrogels at 25 °C.

Gel strength was the inherent structural feature of gelatin-base hydrogels for predicting their physical characteristic. In the previous work, we found that the gel strength had been significantly improved by adding CNCs and mTG into gelatin [25]. In this study, we used APTMS to modify CNCs to introduce amino groups on its surface to form a covalent cross-link between the gelatin chain and CNCs. As shown in Figure 4, the gel strength of the modified CNCs/gelatin hydrogel was slightly improved, and the gel strength of Gel-TG-Si (1.2)-CNCs can reach about 1000 g (25 °C). The gel strength of the Gel-TG-Si-CNCs was not greatly improved as expected. This may be explained by the fact that the addition of CNCs had a greater effect on the gel strength than covalent cross-linking. The interfacial interactions between gelatin chain and CNCs extended the molecular interfacial zones, which successfully transferred mechanical stresses and minimized the crack propagation, and led to the enhancement of gel strength [34], whereas the interfacial interaction types between the two materials, whether chemical and/or physical cross-linking, were relatively less important.

### 2.5. SEM

**Figure 5:**
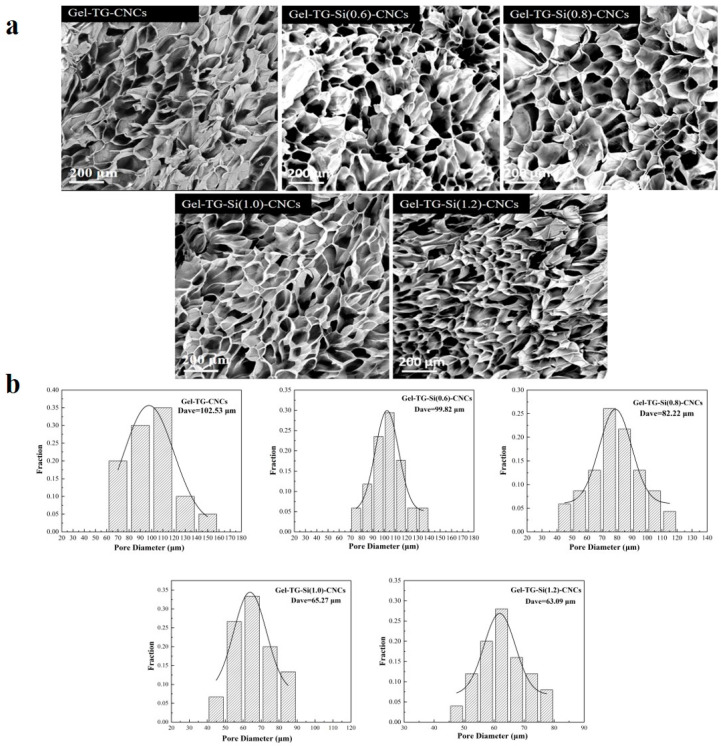
SEM images (**a**) and pore size distributions (**b**) of the freeze-dried hydrogels of Gel-TG-CNCs and Gel-TG-Si-CNCs.

Figure 5a shows the microstructure of Gel-TG-CNCs and Gel-TG-Si-CNCs hydrogels. It can be found that all the gelatin-based hydrogels formed a homogenous three-dimensional network. As reported in our previous study [25], this structure was attributed to the supporting effect of CNCs on the gelatin matrix. All hydrogels presented a cellular structure with smooth walls, and no CNCs can be clearly identified in the pore wall of the scaffold. It was indicated that CNCs were embedded well in the gelatin matrix, which can be attributed to the effective dispersion and the good interfacial compatibility of CNCs [35]. Additionally, the degree of modification of CNCs might have an influence on the pore size of the hydrogels. As displayed in Figure 5b, the pore size distribution gradually tends to be small, narrow and relatively uniform with the increase in modification degree. For lower silanization degree, the samples (Gel-TG-CNCs, Gel-TG-Si (0.6)-CNCs, Gel-TG-Si (0.8)-CNCs) had a relatively loose structure with larger pore size, and their average pore sizes were 102, 100 and 82 μm, respectively. The average pore sizes of Gel-TG-Si (1.0)-CNCs and Gel-TG-Si (1.2)-CNCs hydrogels were significantly smaller (65 μm and 63 μm) in comparison with that of the former three samples. This decrease in pore size was associated with the cross-linking reaction between gelatin chain and CNCs-Si, which enhanced the interaction between the two starting materials and limited the freedom of movement of CNCs-Si, thereby making the structure of the hydrogel more compact.

### 2.6. Biocompatibility Assays

**Figure 6:**
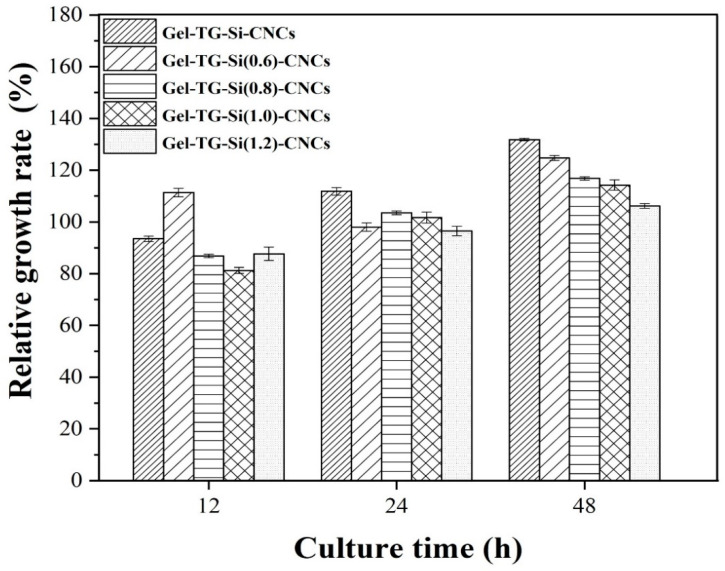
Relative growth rate of HeLa cells results of all hydrogels at different culture time (12 h, 24 h and 48 h).

Biocompatibility is an important criterion for the materials, given their possible application in the biological field. For this reason, the MTT test was performed to evaluate the biocompatibility of the Gel-TG-Si-CNCs hydrogels. The relative growth rate (RGR) of HeLa cells cultured in the leaching solution of all hydrogel samples at different culture times (12 h, 24 h and 48 h) were presented in Figure 6. All the samples yielded cell viability higher than 80% over the incubation period, being considered non-cytotoxic in any hydrogels [36]. It indicates that silanization of CNCs did not affect cell viability. Similar results were reported by Chuah et al. [37] and Vuppaladadium et al. [38], who had determined the cytotoxicity of silanized poly (dimethylsiloxane) (PDMS) and silanized graphene oxide, respectively, and found that the silanized materials did not show any significant toxicity. Notably, cell viability was further increased with increased culture time, indicating the excellent biocompatibility of these gelatin-based hydrogels. This can be attributed to their enzymatic cross-linking via mTG as well as their unique natural and biocompatible composition, gelatin and CNCs.

**Figure 7:**
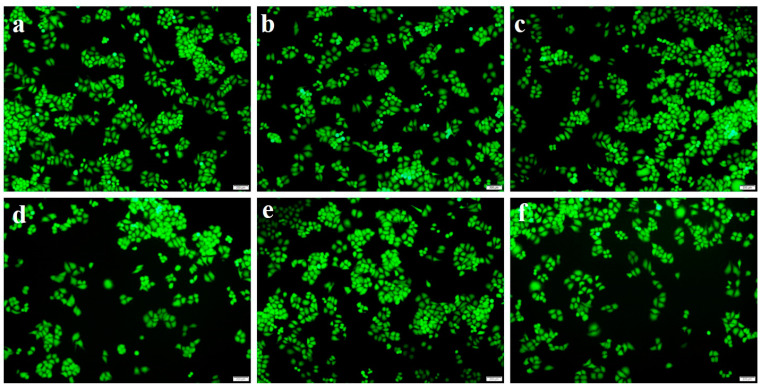
Morphological observation of HeLa cells with FDA staining by fluorescence microscope after culturing for 24 h (scale bar 200 μm); PBS control (**a**); Gel-TG-CNCs (**b**); Gel-TG-Si (0.6)-CNCs (**c**); Gel-TG-Si (0.8)-CNCs(**d**); Gel-TG-Si (1.0)-CNCs (**e**); and Gel-TG-Si (1.2)-CNCs (**f**).

The morphology of HeLa cells after 24 h incubation was observed by a fluorescence microscope using FDA staining, and the images are shown in Figure 7. It was observed that there was no significant change in cell number or cell morphology of the cultured cells. In the case of all examined samples, the cells were distributed uniformly on the culture plate, maintained their original cell morphology in a spindle shape and confirmed as viable due to uptake of green FDA fluorescence. These results were consistent with the results of cell viability detected by MTT. The above results fully demonstrated that the prepared gelatin-based hydrogel material had good biocompatibility, and the introduction of APTMS grafted CNCs did not affect the cytotoxicity. It suggested that the Gel-TG-Si-CNCs hydrogels with excellent biocompatibility were promising for biomedical applications.

## 3. Conclusions

In the research, a series of gelatin-based hydrogels were obtained by enzymatic cross-linking and introducing APTMS grafted CNCs as the strengthening agent. The increase in APTMS amount in the functionalization solution resulted in an increase in the degree of cross-linking between gelatin and grafted CNCs and an improved dispersibility of CNCs within the gelatin matrix. Thus, the prepared hydrogels showed significantly improved mechanical properties and thermo-stability. Most definitely, biocompatibility analysis results using MTT showed that no toxicity is brought upon both enzymatic cross-linking reaction and the introduction of ATMPS grafted CNCs. In conclusion, this paper provides an available reference for preparing mechanically enhanced gelatin-based hydrogels without compromising their biocompatibility, and the as-prepared Gel-TG-Si-CNCs hydrogels were likely to be a promising application in the biomedical field.

## 4. Materials and Methods

### 4.1. Materials

Commercial CNCs solution (solid content 1.5%) was purchased from Zhongshan NanoFC Bio Materials (Guangzhou, China). Type A gelatin (300 bloom), Dulbecco’s Modified Eagle’s Medium (DMEM) and Hela cells were all obtained from the local Sigma-Aldrich supplier (Shanghai, China). Fetal bovine serum (FBS), fluorescein diacetate (FDA) and (3-Aminopropyl) trimethoxysilane (APTMS) (purity ≥97%) were supplied from Macklin Biochemical Co., Ltd. (Shanghai, China). Commercial mTG (200 U/g) was provided by Shanghai Yuanye Bio-Technology Co., Ltd. 3-(4,5-dimethyl-2-thiazolyl)-2,5-diphenyl-2*H*-tetrazolium bromide (MTT) was provided by Thermo Fisher Scientific Inc. (Shanghai, China). Throughout this study, all other chemicals and solvents were of analytical grade or higher, and deionized water was used to prepare all solutions.

### 4.2. Preparation of Gel-TG-Si-CNCs Hydrogels

**Figure 8:**
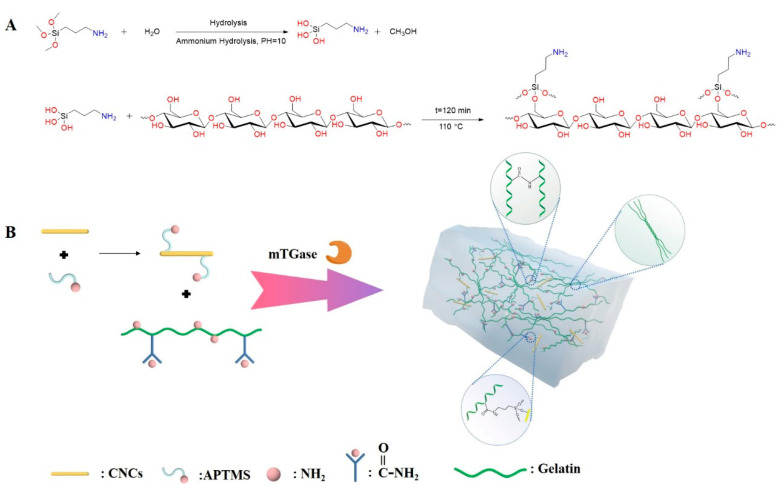
Schematic illustration of APTMS grafted CNCs (**A**) and the fabrication of Gel-TG-Si-CNCs hydrogels (**B**).

Preparation of APTMS grafted CNCs APTMS grafted CNCs (CNCs-Si) samples were prepared as follows. CNCs (20 g) were dispersed in anhydrous ethanol (80 mL) using a high-speed disperser (8000 r/min, 5 min), named solution A. Solution B contained 20 mL deionized water and different volume of APTMS (0.6 mL for CNCs-Si (0.6), 0.8 mL for CNCs-Si (0.8), 1.0 mL for CNCs-Si (1.0) and 1.2 mL for CNCs-Si (1.2). Dissolve 0.6, 0.8, 1.0 and 1.2 mL of APTMS in 20 mL deionized water, respectively. Solution A and B were fully mixed in a hydrothermal reactor, subsequently using ammonia to adjust pH to 10.0 and reacted at 110 °C for 2 h. After the reaction, the mixture was centrifuged (10,000 r/min, 10 min, 4 °C) and washed 3 times with water to remove excess ammonia, 3 times with anhydrous ethanol to remove unreacted silane coupling agent, and 3 times with deionized water to remove residual ethanol. The reaction process of APTMS grafted CNCs is illustrated in Figure 8A.

Preparation of Gel-TG-Si-CNCs The preparation procedure of gelatin/CNCs-Si hydrogels (Gel-TG-Si-CNCs) was illustrated schematically in Figure 8B. Briefly, the gelatin solution (6.67%, *w*/*v*) was prepared by soaking gelatin in deionized water for 2 h and then dissolving it in a water bath at 50 °C. Then, a certain amount of CNCs-Si (2% wt for gelatin) was fully dispersed in deionized water using a high-speed disperser. The two prepared solutions were sufficiently mixed and adjusted pH 6.5 with the NaOH solution of 1.0 mol/L. Subsequently, mTG (10 U/g gelatin) was added into the mixture, which was cross-linked at 50 °C for 60 min and inactivated at 95 °C for 5 min. Based on the different kinds of CNCs-Si, the as-prepared hydrogels were correspondingly named Gel-TG-Si (0.6)-CNCs, Gel-TG-Si (0.8)-CNCs, Gel-TG-Si (1.0)-CNCs and Gel-TG-Si (1.2)-CNCs.

For comparison, gelatin/CNCs hydrogel (Gel-TG-CNCs) was prepared as in our previously published work [25].

### 4.3. Characterization

The CNCs-Si samples were characterized by FTIR Spectroscopy and X-ray photoelectron spectroscopy (XPS). The freeze-dried CNCs and CNCs-Si samples were ground to fine powders with a CryoMill (Retsch, Germany) under liquid nitrogen, and then the ground sample was dried in a vacuum chamber at 80 °C to eliminate any water molecules. FTIR was performed on infrared spectroscopy of IR Prestige-21 (Shimadzu, Japan) using the KBr powder tableting method in the wavenumber range of 400–4000 cm^−1^. XPS measurements were carried out on a Thermo Fisher Scientific ESCALab 250Xi spectrometer (Waltham, MA, USA). Al-K radiation source (1486.6 eV) was used as an X-ray source, and the value of binding energy is calibrated using the C_1s_ peak at 284.8 eV as a reference. The morphology of the as-prepared hydrogels was observed by Phenom Pure^+^ scanning electron microscopy (Thermo Fisher, Eindhoven, The Netherlands) with an acceleration voltage of 10 kV. The pore size distribution was later calculated by the self-contained PoroMetric application. In order to preserve better morphology, before freeze-drying, samples had been cooled down to −80 °C for 24 h in advance. Then, the frozen sample is freeze-dried under vacuum sublimation conditions. The hydrogels were quenched in liquid nitrogen followed by freeze-drying.

### 4.4. Rheological Behavior

The ARES/G2 rotational rheometer (TA Instruments, New Castle, DE, USA) was employed to analyze the rheological behavior of the as-prepared Gel-TG-CNCs and Gel-TG-Si-CNCs hydrogels. The parallel plate instrument of the rheometer was used with a diameter of 25 mm and a gap size of 1.0 mm. Temperature sweeps were performed by heating from 5 °C to 40 °C and then cooling from 40 °C to 5 °C at a rate of 0.5 °C per min (holding at 5 °C or 40 °C for 3 min), at a fixed frequency of 1 Hz, with the constant strain of 0.5%. The dynamic frequency scanning was carried out at 5 °C and 15 °C with angular frequency ranging from 0.0628 to 62.8 rad s^−1^ and constant strain of 0.5%. The storage modulus value (G′) at different frequencies was recorded, and all assays were accomplished within the linear viscoelastic region.

### 4.5. Gel Strength Test

The TA.XT plus texture analyzer (Stable Micro System, Surrey, UK) was used to determine the gel strength of the as-prepared hydrogels with a cylinder probe of P/0.5. Before testing, samples were stored for stabilization at 5 °C for 24 h. For the compression procedure, a 5 kN load cell was used with a speed of 1.5 mm/min at 25 °C.

### 4.6. Biocompatibility Assays

Referred to the published study of Cui et al., the biocompatibility of the as-prepared hydrogels was evaluated by Hela cells [30]. Briefly, hydrogels were firstly freeze-dried, then ground to fine powders and sterilized with UV light for 2 h. Then, 30 mg sterilized hydrogels powders were dispersed into 5 mL PBS buffer (0.01 M, pH 7.4), and the leaching solutions were collected by filtering with a 0.45 µm filter after 12 h. The Hela cells were inoculated at 37 °C in 96-well plates with the seeding density of 8 × 10^3^ cells/well for different time intervals (12 h, 24 h and 48 h), after individual adding 20 mL leaching solution and DMEM containing 10% FBS. The optical density (OD) value of the culture solution was measured by Spectra Max M5 microplate reader (Molecular Devices, Los Angeles, CA, USA) at 570 nm, and the cell viability was calculated by normalization to the intensity of the control, which was cells in PBS buffer plus DMEM containing 10% FBS. In addition, cells cultured for 24 h were stained with Fluorescein diacetate (FDA) solution (20 µg/mL) and observed under a fluorescence microscope (Olympus U-RFL-T, Tokyo, Japan) to evaluate cell growth.

## Figures and Tables

**Table 1:** The atomic concentration and O/C ratio of CNCs and CNCs-Si.

	C_1s_	O_1s_	N_1s_	Si_2p_	O/C
CNCs	60.36	39.64			0.66
CNCs–Si	59.03	38.01	1.42	1.54	0.64

## Data Availability

Not applicable.
